# Dibromidodi-μ-hydroxido-di-μ_3_-oxido-octa­phenyl­tetra­tin(IV)

**DOI:** 10.1107/S1600536809051691

**Published:** 2009-12-04

**Authors:** Quai Ling Yap, Kong Mun Lo, Seik Weng Ng

**Affiliations:** a Department of Chemistry, University of Malaya, 50603 Kuala Lumpur, Malaysia

## Abstract

In the centrosymmetric title compound, [Sn_4_Br_2_(C_6_H_5_)_8_O_2_(OH)_2_], the four tin(IV) atoms are bridged by the hydroxo and oxo ligands, forming a ladder-like array of three edge-connected Sn_2_O_2_ squares. The two independent tin atoms show distorted trigonal-bipyramidal SnC_2_O_3_ and SnC_2_O_2_Br coordination geometries.

## Related literature

For other [Sn_4_ *X*_2_(O)_2_(OH)_2_ *R*_8_] (*X* = halogen, *R* = organic group) structures, see: Baumeister *et al.* (2002 ▶); Beckmann *et al.* (2001 ▶); Cox & Tiekink (1994 ▶); Kresinski *et al.* (1994 ▶); Lo & Ng (2009 ▶); Mohamed *et al.* (2004 ▶); Puff *et al.* (1983 ▶); Tiekink (1991 ▶); Vollano *et al.* (1984 ▶).
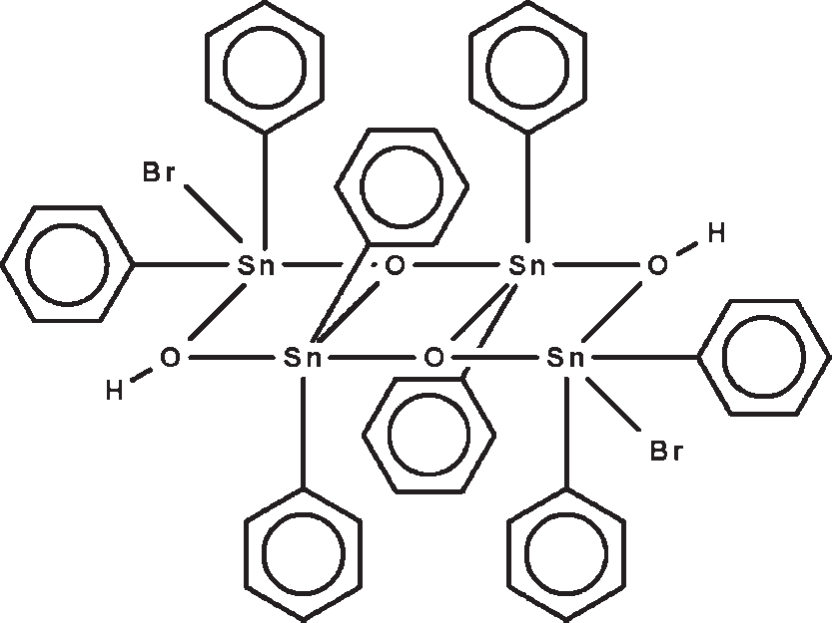


## Experimental

### 

#### Crystal data

[Sn_4_Br_2_(C_6_H_5_)_8_O_2_(OH)_2_]
*M* *_r_* = 1317.40
Triclinic, 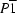

*a* = 10.3759 (1) Å
*b* = 10.8247 (1) Å
*c* = 12.0341 (2) Å
α = 77.1809 (7)°
β = 65.4426 (7)°
γ = 75.2040 (7)°
*V* = 1178.26 (3) Å^3^
*Z* = 1
Mo *K*α radiation
μ = 3.83 mm^−1^
*T* = 293 K
0.25 × 0.25 × 0.25 mm

#### Data collection

Bruker SMART APEX diffractometer
Absorption correction: multi-scan (*SADABS*; Sheldrick, 1996 ▶) *T*_min_ = 0.539, *T*_max_ = 0.746
10751 measured reflections
5309 independent reflections
4449 reflections with *I* > 2σ(*I*)
*R*_int_ = 0.028

#### Refinement

*R*[*F* ^2^ > 2σ(*F* ^2^)] = 0.042
*wR*(*F* ^2^) = 0.160
*S* = 1.06
5309 reflections
262 parameters
H-atom parameters constrained
Δρ_max_ = 2.39 e Å^−3^
Δρ_min_ = −1.38 e Å^−3^

### 

Data collection: *APEX2* software (Bruker, 2008 ▶); cell refinement: *SAINT* (Bruker, 2008 ▶); data reduction: *SAINT*; program(s) used to solve structure: *SHELXS97* (Sheldrick, 2008 ▶); program(s) used to refine structure: *SHELXL97* (Sheldrick, 2008 ▶); molecular graphics: *X-SEED* (Barbour, 2001 ▶); software used to prepare material for publication: *publCIF* (Westrip, 2009 ▶).

## Supplementary Material

Crystal structure: contains datablocks global, I. DOI: 10.1107/S1600536809051691/hb5234sup1.cif

Structure factors: contains datablocks I. DOI: 10.1107/S1600536809051691/hb5234Isup2.hkl

Additional supplementary materials:  crystallographic information; 3D view; checkCIF report

## Figures and Tables

**Table 1:** Selected bond lengths (Å)

Sn1—O1^i^	2.068 (4)
Sn1—O1	2.095 (4)
Sn1—C1	2.117 (6)
Sn1—C7	2.120 (6)
Sn1—O2	2.171 (4)
Sn2—O1^i^	2.029 (4)
Sn2—C13	2.118 (7)
Sn2—C19	2.126 (7)
Sn2—O2	2.167 (4)
Sn2—Br1	2.6444 (9)

Symmetry code: (i) 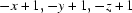.

**Fig. 1.**
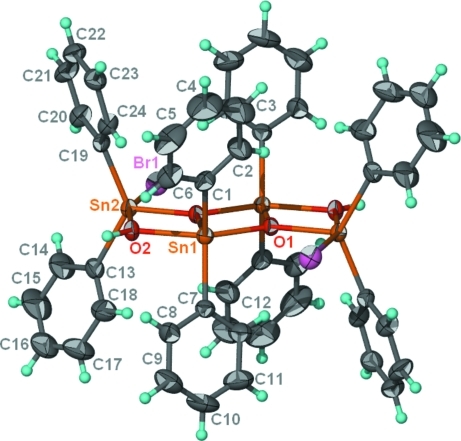
View of (I) at the 50% probability level; hydrogen atoms are drawn as spheres of arbitrary radius. Unlabelled atoms are generated by the symmetry operation (1–x, 1–y, 1–z).

## supplementary crystallographic information

### Experimental

In an attempt to cleave a tin-carbon bond in a tetraorganotin compound, bis(3-methyl-2-thienyl)diphenyltin (0.47 g,1 mmol) and trimethylphenylammonium tribromide (0.38 g, 1 mmol) were heated in ethanol for three hours. Colourless blocks of (I) separated from solution after a few days.

### Refinement

Carbon- and oxygen-bound H-atoms were placed in calculated positions (C–H 0.93 Å) and were included in the refinement in the riding model approximation, with *U*(H) set to 1.2*U*(C,*O*).

The final difference Fourier map had a peak near Sn1 and a hole near Sn2.

### Crystal data

**Table:**

[Sn_4_Br_2_(C_6_H_5_)_8_O_2_(OH)_2_]	*Z* = 1
*M**_r_* = 1317.40	*F*(000) = 632
Triclinic, *P*1	*D*_x_ = 1.857 Mg m^−^^3^
Hall symbol: -P 1	Mo *K*α radiation, λ = 0.71073 Å
*a* = 10.3759 (1) Å	Cell parameters from 7354 reflections
*b* = 10.8247 (1) Å	θ = 2.2–28.5°
*c* = 12.0341 (2) Å	µ = 3.83 mm^−^^1^
α = 77.1809 (7)°	*T* = 293 K
β = 65.4426 (7)°	Block, colourless
γ = 75.2040 (7)°	0.25 × 0.25 × 0.25 mm
*V* = 1178.26 (3) Å^3^	

### Data collection

**Table:**

Bruker SMART APEX diffractometer	5309 independent reflections
Radiation source: fine-focus sealed tube	4449 reflections with *I* > 2σ(*I*)
graphite	*R*_int_ = 0.028
ω scans	θ_max_ = 27.5°, θ_min_ = 1.9°
Absorption correction: multi-scan (*SADABS*; Sheldrick, 1996)	*h* = −13→13
*T*_min_ = 0.539, *T*_max_ = 0.746	*k* = −14→13
10751 measured reflections	*l* = −15→15

### Refinement

**Table:**

Refinement on *F*^2^	Primary atom site location: structure-invariant direct methods
Least-squares matrix: full	Secondary atom site location: difference Fourier map
*R*[*F*^2^ > 2σ(*F*^2^)] = 0.042	Hydrogen site location: inferred from neighbouring sites
*wR*(*F*^2^) = 0.160	H-atom parameters constrained
*S* = 1.06	*w* = 1/[σ^2^(*F*_o_^2^) + (0.0952*P*)^2^ + 4.7584*P*] where *P* = (*F*_o_^2^ + 2*F*_c_^2^)/3
5309 reflections	(Δ/σ)_max_ = 0.001
262 parameters	Δρ_max_ = 2.38 e Å^−^^3^
0 restraints	Δρ_min_ = −1.38 e Å^−^^3^

### Fractional atomic coordinates and isotropic or equivalent isotropic displacement parameters (Å^2^)

**Table:**

	*x*	*y*	*z*	*U*_iso_*/*U*_eq_	
Sn1	0.38584 (4)	0.45011 (4)	0.46172 (4)	0.03147 (14)	
Sn2	0.52171 (5)	0.20525 (4)	0.63535 (4)	0.03495 (15)	
Br1	0.70321 (9)	0.23768 (8)	0.72424 (7)	0.0508 (2)	
O1	0.4796 (5)	0.6078 (4)	0.4416 (4)	0.0315 (8)	
O2	0.3774 (5)	0.2494 (4)	0.5377 (4)	0.0364 (9)	
H2O	0.3312	0.2022	0.5311	0.044*	
C1	0.4869 (8)	0.4083 (7)	0.2777 (6)	0.0437 (15)	
C2	0.6000 (10)	0.4680 (9)	0.1951 (7)	0.062 (2)	
H2	0.6262	0.5303	0.2198	0.074*	
C3	0.6748 (14)	0.4366 (14)	0.0765 (9)	0.092 (4)	
H3	0.7537	0.4739	0.0230	0.111*	
C4	0.6308 (17)	0.3499 (15)	0.0393 (11)	0.104 (5)	
H4	0.6786	0.3303	−0.0410	0.125*	
C5	0.5169 (15)	0.2913 (12)	0.1190 (11)	0.088 (4)	
H5	0.4865	0.2338	0.0921	0.106*	
C6	0.4471 (11)	0.3183 (9)	0.2404 (8)	0.060 (2)	
H6	0.3736	0.2753	0.2959	0.072*	
C7	0.1737 (7)	0.5207 (7)	0.5813 (6)	0.0379 (13)	
C8	0.0633 (8)	0.4546 (8)	0.6167 (8)	0.0524 (18)	
H8	0.0782	0.3810	0.5819	0.063*	
C9	−0.0724 (10)	0.4978 (12)	0.7057 (10)	0.075 (3)	
H9	−0.1485	0.4550	0.7284	0.090*	
C10	−0.0900 (10)	0.6034 (13)	0.7578 (9)	0.082 (3)	
H10	−0.1780	0.6296	0.8196	0.098*	
C11	0.0158 (11)	0.6711 (12)	0.7228 (11)	0.084 (3)	
H11	−0.0008	0.7451	0.7576	0.100*	
C12	0.1506 (9)	0.6298 (9)	0.6340 (8)	0.0554 (19)	
H12	0.2247	0.6754	0.6103	0.067*	
C13	0.3559 (8)	0.1680 (7)	0.8092 (6)	0.0453 (15)	
C14	0.3843 (12)	0.0721 (11)	0.8954 (9)	0.077 (3)	
H14	0.4784	0.0286	0.8804	0.093*	
C15	0.2745 (16)	0.0389 (14)	1.0052 (10)	0.103 (4)	
H15	0.2949	−0.0282	1.0623	0.123*	
C16	0.1375 (15)	0.1039 (13)	1.0295 (10)	0.091 (4)	
H16	0.0637	0.0796	1.1022	0.109*	
C17	0.1077 (13)	0.2043 (14)	0.9484 (10)	0.094 (4)	
H17	0.0144	0.2512	0.9670	0.113*	
C18	0.2175 (10)	0.2370 (10)	0.8367 (8)	0.065 (2)	
H18	0.1971	0.3056	0.7809	0.078*	
C19	0.6770 (7)	0.0737 (6)	0.5153 (6)	0.0388 (13)	
C20	0.7810 (9)	−0.0145 (8)	0.5494 (8)	0.0527 (18)	
H20	0.7787	−0.0227	0.6288	0.063*	
C21	0.8893 (9)	−0.0909 (9)	0.4642 (9)	0.062 (2)	
H21	0.9578	−0.1515	0.4879	0.074*	
C22	0.8961 (9)	−0.0784 (8)	0.3475 (9)	0.059 (2)	
H22	0.9705	−0.1285	0.2911	0.071*	
C23	0.7926 (9)	0.0089 (8)	0.3123 (8)	0.0541 (19)	
H23	0.7975	0.0177	0.2321	0.065*	
C24	0.6816 (9)	0.0834 (7)	0.3962 (7)	0.0495 (17)	
H24	0.6101	0.1399	0.3732	0.059*	

### Atomic displacement parameters (Å^2^)

**Table:**

	*U*^11^	*U*^22^	*U*^33^	*U*^12^	*U*^13^	*U*^23^
Sn1	0.0317 (2)	0.0315 (2)	0.0317 (2)	−0.00616 (16)	−0.01214 (17)	−0.00462 (15)
Sn2	0.0381 (2)	0.0287 (2)	0.0345 (2)	−0.00560 (17)	−0.01235 (18)	−0.00091 (16)
Br1	0.0555 (4)	0.0517 (4)	0.0531 (4)	−0.0067 (3)	−0.0305 (3)	−0.0063 (3)
O1	0.033 (2)	0.026 (2)	0.036 (2)	−0.0070 (16)	−0.0157 (17)	0.0008 (16)
O2	0.040 (2)	0.030 (2)	0.045 (2)	−0.0123 (18)	−0.0197 (19)	−0.0005 (17)
C1	0.055 (4)	0.039 (3)	0.033 (3)	0.000 (3)	−0.017 (3)	−0.008 (3)
C2	0.068 (5)	0.070 (6)	0.040 (4)	−0.024 (5)	−0.006 (4)	−0.010 (4)
C3	0.104 (9)	0.114 (10)	0.041 (5)	−0.033 (8)	−0.002 (5)	−0.014 (5)
C4	0.127 (11)	0.120 (11)	0.050 (6)	−0.001 (9)	−0.019 (7)	−0.036 (6)
C5	0.123 (10)	0.082 (7)	0.080 (7)	0.005 (7)	−0.055 (7)	−0.046 (6)
C6	0.069 (5)	0.061 (5)	0.061 (5)	−0.005 (4)	−0.032 (4)	−0.024 (4)
C7	0.031 (3)	0.041 (3)	0.038 (3)	−0.005 (3)	−0.012 (2)	−0.004 (3)
C8	0.039 (4)	0.049 (4)	0.066 (5)	−0.010 (3)	−0.017 (3)	−0.005 (3)
C9	0.044 (5)	0.096 (8)	0.077 (6)	−0.024 (5)	−0.016 (4)	0.002 (6)
C10	0.044 (5)	0.114 (9)	0.061 (6)	−0.008 (5)	0.006 (4)	−0.018 (6)
C11	0.058 (6)	0.086 (8)	0.090 (7)	−0.003 (5)	−0.001 (5)	−0.046 (6)
C12	0.042 (4)	0.059 (5)	0.060 (5)	−0.011 (4)	−0.007 (3)	−0.021 (4)
C13	0.051 (4)	0.043 (4)	0.038 (3)	−0.016 (3)	−0.010 (3)	−0.003 (3)
C14	0.075 (6)	0.073 (6)	0.059 (5)	−0.011 (5)	−0.019 (5)	0.023 (5)
C15	0.110 (10)	0.107 (10)	0.058 (6)	−0.033 (8)	−0.015 (6)	0.033 (6)
C16	0.101 (9)	0.094 (8)	0.049 (5)	−0.038 (7)	0.010 (5)	−0.009 (5)
C17	0.069 (7)	0.115 (10)	0.065 (6)	−0.025 (7)	0.016 (5)	−0.024 (7)
C18	0.058 (5)	0.068 (6)	0.053 (5)	−0.009 (4)	−0.009 (4)	−0.005 (4)
C19	0.036 (3)	0.030 (3)	0.050 (4)	−0.007 (3)	−0.016 (3)	−0.008 (3)
C20	0.054 (4)	0.046 (4)	0.060 (5)	−0.002 (3)	−0.025 (4)	−0.012 (3)
C21	0.051 (5)	0.048 (5)	0.087 (6)	0.011 (4)	−0.031 (4)	−0.024 (4)
C22	0.040 (4)	0.051 (5)	0.081 (6)	−0.003 (3)	−0.013 (4)	−0.023 (4)
C23	0.054 (4)	0.058 (5)	0.048 (4)	−0.018 (4)	−0.009 (3)	−0.014 (3)
C24	0.052 (4)	0.038 (4)	0.055 (4)	−0.002 (3)	−0.019 (3)	−0.010 (3)

### Geometric parameters (Å, °)

**Table:**

Sn1—O1^i^	2.068 (4)	C9—H9	0.9300
Sn1—O1	2.095 (4)	C10—C11	1.345 (16)
Sn1—C1	2.117 (6)	C10—H10	0.9300
Sn1—C7	2.120 (6)	C11—C12	1.396 (12)
Sn1—O2	2.171 (4)	C11—H11	0.9300
Sn2—O1^i^	2.029 (4)	C12—H12	0.9300
Sn2—C13	2.118 (7)	C13—C14	1.365 (11)
Sn2—C19	2.126 (7)	C13—C18	1.377 (12)
Sn2—O2	2.167 (4)	C14—C15	1.385 (14)
Sn2—Br1	2.6444 (9)	C14—H14	0.9300
O1—Sn2^i^	2.029 (4)	C15—C16	1.355 (18)
O1—Sn1^i^	2.068 (4)	C15—H15	0.9300
O2—H2O	0.8200	C16—C17	1.356 (19)
C1—C6	1.369 (11)	C16—H16	0.9300
C1—C2	1.384 (11)	C17—C18	1.397 (13)
C2—C3	1.384 (12)	C17—H17	0.9300
C2—H2	0.9300	C18—H18	0.9300
C3—C4	1.365 (19)	C19—C20	1.383 (10)
C3—H3	0.9300	C19—C24	1.394 (11)
C4—C5	1.37 (2)	C20—C21	1.393 (11)
C4—H4	0.9300	C20—H20	0.9300
C5—C6	1.395 (13)	C21—C22	1.355 (13)
C5—H5	0.9300	C21—H21	0.9300
C6—H6	0.9300	C22—C23	1.381 (12)
C7—C12	1.384 (10)	C22—H22	0.9300
C7—C8	1.373 (10)	C23—C24	1.385 (11)
C8—C9	1.407 (12)	C23—H23	0.9300
C8—H8	0.9300	C24—H24	0.9300
C9—C10	1.360 (16)		
			
O1^i^—Sn1—O1	73.05 (18)	C9—C8—H8	119.9
O1^i^—Sn1—C1	113.5 (2)	C10—C9—C8	118.7 (9)
O1—Sn1—C1	98.9 (2)	C10—C9—H9	120.6
O1^i^—Sn1—C7	109.8 (2)	C8—C9—H9	120.6
O1—Sn1—C7	98.4 (2)	C11—C10—C9	122.1 (9)
C1—Sn1—C7	136.4 (3)	C11—C10—H10	118.9
O1^i^—Sn1—O2	72.57 (17)	C9—C10—H10	118.9
O1—Sn1—O2	145.58 (17)	C10—C11—C12	119.7 (10)
C1—Sn1—O2	93.1 (2)	C10—C11—H11	120.2
C7—Sn1—O2	94.7 (2)	C12—C11—H11	120.2
O1^i^—Sn2—C13	116.9 (2)	C7—C12—C11	119.8 (8)
O1^i^—Sn2—C19	113.2 (2)	C7—C12—H12	120.1
C13—Sn2—C19	129.4 (3)	C11—C12—H12	120.1
O1^i^—Sn2—O2	73.38 (17)	C14—C13—C18	118.6 (8)
C13—Sn2—O2	93.3 (2)	C14—C13—Sn2	120.1 (7)
C19—Sn2—O2	94.0 (2)	C18—C13—Sn2	121.3 (6)
O1^i^—Sn2—Br1	86.33 (12)	C13—C14—C15	120.8 (10)
C13—Sn2—Br1	95.6 (2)	C13—C14—H14	119.6
C19—Sn2—Br1	94.25 (18)	C15—C14—H14	119.6
O2—Sn2—Br1	159.71 (12)	C16—C15—C14	120.1 (11)
Sn2^i^—O1—Sn1^i^	111.45 (19)	C16—C15—H15	120.0
Sn2^i^—O1—Sn1	141.6 (2)	C14—C15—H15	120.0
Sn1^i^—O1—Sn1	106.95 (18)	C17—C16—C15	120.3 (10)
Sn2—O2—Sn1	102.60 (18)	C17—C16—H16	119.9
Sn2—O2—H2O	128.7	C15—C16—H16	119.9
Sn1—O2—H2O	128.7	C16—C17—C18	119.8 (12)
C6—C1—C2	119.3 (7)	C16—C17—H17	120.1
C6—C1—Sn1	121.1 (6)	C18—C17—H17	120.1
C2—C1—Sn1	119.5 (6)	C13—C18—C17	120.2 (10)
C3—C2—C1	121.1 (9)	C13—C18—H18	119.9
C3—C2—H2	119.5	C17—C18—H18	119.9
C1—C2—H2	119.5	C20—C19—C24	119.3 (7)
C4—C3—C2	119.0 (11)	C20—C19—Sn2	121.2 (5)
C4—C3—H3	120.5	C24—C19—Sn2	119.4 (5)
C2—C3—H3	120.5	C19—C20—C21	119.6 (8)
C3—C4—C5	120.9 (10)	C19—C20—H20	120.2
C3—C4—H4	119.6	C21—C20—H20	120.2
C5—C4—H4	119.6	C22—C21—C20	121.0 (8)
C4—C5—C6	119.7 (11)	C22—C21—H21	119.5
C4—C5—H5	120.1	C20—C21—H21	119.5
C6—C5—H5	120.1	C21—C22—C23	119.9 (8)
C1—C6—C5	119.9 (10)	C21—C22—H22	120.0
C1—C6—H6	120.0	C23—C22—H22	120.0
C5—C6—H6	120.0	C24—C23—C22	120.1 (8)
C12—C7—C8	119.5 (7)	C24—C23—H23	119.9
C12—C7—Sn1	118.4 (5)	C22—C23—H23	119.9
C8—C7—Sn1	121.8 (5)	C23—C24—C19	120.0 (7)
C7—C8—C9	120.1 (9)	C23—C24—H24	120.0
C7—C8—H8	119.9	C19—C24—H24	120.0
			
O1^i^—Sn1—O1—Sn2^i^	−177.8 (5)	C12—C7—C8—C9	0.2 (12)
C1—Sn1—O1—Sn2^i^	70.2 (4)	Sn1—C7—C8—C9	173.6 (7)
C7—Sn1—O1—Sn2^i^	−69.5 (4)	C7—C8—C9—C10	−1.9 (15)
O2—Sn1—O1—Sn2^i^	179.3 (3)	C8—C9—C10—C11	3.2 (19)
O1^i^—Sn1—O1—Sn1^i^	0.0	C9—C10—C11—C12	−3(2)
C1—Sn1—O1—Sn1^i^	−112.0 (2)	C8—C7—C12—C11	0.2 (13)
C7—Sn1—O1—Sn1^i^	108.2 (2)	Sn1—C7—C12—C11	−173.4 (8)
O2—Sn1—O1—Sn1^i^	−2.9 (4)	C10—C11—C12—C7	1.0 (17)
O1^i^—Sn2—O2—Sn1	−0.23 (16)	O1^i^—Sn2—C13—C14	−143.7 (8)
C13—Sn2—O2—Sn1	116.9 (3)	C19—Sn2—C13—C14	45.3 (9)
C19—Sn2—O2—Sn1	−113.2 (2)	O2—Sn2—C13—C14	143.2 (8)
Br1—Sn2—O2—Sn1	0.7 (5)	Br1—Sn2—C13—C14	−55.0 (8)
O1^i^—Sn1—O2—Sn2	0.23 (16)	O1^i^—Sn2—C13—C18	37.9 (8)
O1—Sn1—O2—Sn2	3.2 (4)	C19—Sn2—C13—C18	−133.2 (7)
C1—Sn1—O2—Sn2	113.9 (3)	O2—Sn2—C13—C18	−35.2 (7)
C7—Sn1—O2—Sn2	−109.1 (2)	Br1—Sn2—C13—C18	126.6 (7)
O1^i^—Sn1—C1—C6	112.1 (6)	C18—C13—C14—C15	4.3 (17)
O1—Sn1—C1—C6	−172.6 (6)	Sn2—C13—C14—C15	−174.2 (11)
C7—Sn1—C1—C6	−60.4 (8)	C13—C14—C15—C16	−2(2)
O2—Sn1—C1—C6	39.7 (7)	C14—C15—C16—C17	−2(2)
O1^i^—Sn1—C1—C2	−64.6 (7)	C15—C16—C17—C18	3(2)
O1—Sn1—C1—C2	10.7 (7)	C14—C13—C18—C17	−3.3 (16)
C7—Sn1—C1—C2	122.9 (7)	Sn2—C13—C18—C17	175.2 (9)
O2—Sn1—C1—C2	−137.0 (7)	C16—C17—C18—C13	−0.3 (19)
C6—C1—C2—C3	−1.1 (15)	O1^i^—Sn2—C19—C20	123.9 (6)
Sn1—C1—C2—C3	175.6 (9)	C13—Sn2—C19—C20	−64.8 (7)
C1—C2—C3—C4	3.3 (19)	O2—Sn2—C19—C20	−162.4 (6)
C2—C3—C4—C5	−2(2)	Br1—Sn2—C19—C20	36.1 (6)
C3—C4—C5—C6	−1(2)	O1^i^—Sn2—C19—C24	−50.8 (6)
C2—C1—C6—C5	−2.4 (13)	C13—Sn2—C19—C24	120.5 (6)
Sn1—C1—C6—C5	−179.1 (8)	O2—Sn2—C19—C24	22.9 (6)
C4—C5—C6—C1	3.7 (17)	Br1—Sn2—C19—C24	−138.6 (6)
O1^i^—Sn1—C7—C12	63.6 (6)	C24—C19—C20—C21	0.7 (12)
O1—Sn1—C7—C12	−11.3 (6)	Sn2—C19—C20—C21	−174.0 (6)
C1—Sn1—C7—C12	−123.7 (6)	C19—C20—C21—C22	1.4 (14)
O2—Sn1—C7—C12	136.8 (6)	C20—C21—C22—C23	−1.7 (14)
O1^i^—Sn1—C7—C8	−109.8 (6)	C21—C22—C23—C24	−0.2 (13)
O1—Sn1—C7—C8	175.3 (6)	C22—C23—C24—C19	2.3 (12)
C1—Sn1—C7—C8	62.9 (7)	C20—C19—C24—C23	−2.5 (12)
O2—Sn1—C7—C8	−36.7 (6)	Sn2—C19—C24—C23	172.3 (6)

Symmetry codes: (i) −*x*+1, −*y*+1, −*z*+1.
